# Cyperotundone promotes chemosensitivity of breast cancer via SRSF1

**DOI:** 10.3389/fphar.2025.1510161

**Published:** 2025-03-19

**Authors:** Chao Li, Lin Wang, Zhaoyun Liu, Xinzhao Wang, Luhao Sun, Xiang Song, Zhiyong Yu

**Affiliations:** ^1^ Shandong University Cancer Center, Jinan, Shandong, China; ^2^ Breast Cancer Center, Shandong Cancer Hospital and Institute, Shandong First Medical University and Shandong Academy of Medical Sciences, Jinan, Shandong, China; ^3^ Breast Disease Center, The Affiliated Hospital of Qingdao University, Qingdao, Shandong, China

**Keywords:** breast cancer, drug resistance, EMT, cyperotundone, SRSF1

## Abstract

Breast cancer is among the most common malignancies and the leading cause of cancer-related deaths in women. SRSF1 proteins belong to an important splicing factor (SF) family and bind to different splicing regulatory elements (SREs) to promote or inhibit splicing, such as oncogenic splice-switching of PTpMT1, which promoting the progression of cancer. Cyperotundone (CYT) is the major bioactive component of sedge and reported to exhibit multiple biological functions, including its potent cytotoxic effect on breast cancer cells. However, the detailed impact and molecular mechanisms of CYT in breast cancer remain poorly understood. This study aimed to investigate the effects of CYT on breast cancer drug resistance and to explore the molecular mechanisms. CYT significantly suppressed the *in vitro* and *in vivo* growth of BC cells without affecting the normal cells at different doses (P < 0.001), induced cell apoptosis, and inhibited the migration and invasion of drug-resistant BC. In comparison with the mono treatment with CYT, combination of CYT and doxorubicin (Dox) enhanced the effects. CYT treatment regulated the RNA and protein levels of epithelial mesenchymal transition (EMT) biomarkers, suppressed the sphere formation ability and expression of cancer stem cell biomarkers in drug resistant BC cells. Results from transcriptome sequencing analysis and experiments identified significantly decreased SRSF1 level in drug resistant cells after CYT treatment. RNA and protein levels of SRSF1 and MYO1B were higher in drug resistant BC cells (P < 0.01). SRSF1 regulated alternative splicing of MYO1B to enhance the ability of drug resistance. Knockdown of SRSF1 significantly decreased expression of full-length MYO1B protein in drug-resistant BC cells (*P* < 0.05). Overexpression of SRSF1 and MYO1B revered the inhibitory effects of CYT. In conclusion, CYT repressed the growth and metastasis of BC cells and recovered drug sensitivity, through SRSF1-regulated the alternative splicing of MYO1B RNAs, which may represent a novel molecular mechanism to overcome drug resistance in breast cancer. Targeting SRSF1 or MYO1B may be identified as a novel molecular mechanism to against drug resistant in breast cancer.

## 1 Introduction

Breast cancer (BC) is one of the most common malignancies and ranks as the second leading cause of cancer-related deaths in women (Khongkow et al., 2013). Despite the effects of surgical intervention, chemotherapy and radiotherapy, the frequently developed chemotherapy resistance and advanced metastasis lead to high mortality and poor prognosis of BC (Ma et al., 2021; Zhang et al., 2021). A deep understanding of the molecular mechanisms underlying drug resistance is urgent and essential to improve the therapeutic efficacy and survival of patients with advanced BC.

Alternative splicing (AS) is a post-transcriptional process that widely exists in genes and plays a critical role in expanding the transcript and protein diversity, and is increasingly recognized for its role in cancer progression and therapy resistance (Baralle and Giudice, 2017; Bergsma et al., 2018). In breast cancer (BC), AS events contribute to disease progression and metastasis, which are key determinants of patient survival (Bhadra et al., 2020; Bonnal et al., 2020). Abnormal AS can lead to the production of protein isoforms that promote cell growth, resistance to apoptosis, and increased metastatic potential, all of which are critical for the aggressiveness of BC (Zheng et al., 2020; Cherry and Lynch, 2020; Liu and Rabadan, 2021). While AS has been extensively studied for its role in therapeutic resistance, the contribution of splicing factors (SFs), such as SRSF1, to BC progression and drug resistance is gaining increasing recognition and deserves further investigation.

SRSF1, a key SF, binds to different splicing regulatory elements (SREs), such as exonic splicing enhancers (ESEs) and intronic splicing enhancers (ISEs), to regulate AS by promoting or inhibiting the recognition of splice sites (Paz et al., 2021). In addition, SRSF1 performs other biological functions, including transcriptional activation, RNA stabilization, mRNA transport, and translation control (Paz et al., 2021). Notably, the carcinogenic effects of SRSF1 and SRSF1-mediated AS events have been reported in BC. Our early study also demonstrated that SRSF1 reduces cisplatin chemosensitivity of triple-negative BC cells through the circSEPT9/GCH1 axis. Besides, SRSF1 promotes BC progression via oncogenic splice switching of PTpMT1 and PRMT1-mediated SRSF1 methylation could suppress oncogenic exon inclusion events and breast tumorigenesis (Du et al., 2021; Shao et al., 2023). However, its clinical significance, specific targets and detailed regulatory mechanisms in BC remain unclear.

Exploring the molecular mechanisms of SRSF1 in breast cancer, as above mentioned, may help to identify a new target to chemotherapy resistance, finding drugs to overcome drug resistance could explore a potential therapeutic approach to solve the clinical problem. Our early findings had demonstrated the anticancer activity of Cyperus rotundus ethanol extract (EECR) on triple-negative breast cancer. The cyperus rotundus belongs to the sedge family and has been widely reported in pharmacological studies (Ribeiro et al., 2019). As an important source of traditional medicine, sedges have been reported as potential treatments for a variety of diseases (Ribeiro et al., 2019). Cyperotundone (CYT) is the main active component of sedge and exhibits multiple biological functions such as anti-inflammatory, anti-oxidation, antibacterial, neuroprotective, anti-cancer, anti-depression, anti-obesity, anti-arthritis, vasodilatation, bronchiectasis, spasmolysis and estrogen (Shao et al., 2023; Wang et al., 2019). Recent studies have suggested that CYT may play a significant role in overcoming drug resistance in BC by modulating processes involved in oxidative stress and apoptosis. In particular, CYT has been shown to enhance the chemosensitivity of BC cells to conventional chemotherapeutic agents, suggesting its potential as a therapeutic agent to combat drug resistance in BC. However, the specific role and underlying molecular mechanisms of CYT in BC remain unclear. In this study, we explored the effects of CYT on drug resistance of BC and investigated the correlated molecular mechanisms.

## 2 Material and methods

### 2.1 Cell culture

Human breast cancer cell lines MCF7, MDA-MB-231 and human normal mammary epithelial cells MCF10A were cultured in MEM medium (Hyclone, SH30024.01B) with 1% streptomycin and penicillin (Sigma) and 10% fetal bovine serum (Gibco) at 37 °C in a humidified incubator with 95% air and 5% CO_2_. Drug-resistant breast cancer cell lines MCF7-DR and MDA-MB-231-DR were cultured in MEM medium containing low concentration of doxorubicin (DOX, 0.1 µM; MCE). All cells were cultured in a 37°C incubator containing 5% CO_2_.

### 2.2 Cell treatment and transfection

The cells were digested and seeded into 6-well plates at a density of 10^6^ cells/well. After attachment, the cells were starved in serum-free medium for 2 h, then the mixture of Lipofectamine 2000 (Invitrogen, 15 µL) and plasmids 6 µg or siRNAs 200 pmol (Biomed) was added to incubate for 8 h. The medium was changed to complete and incubated for another 40 h. Cells were then collected and used for following experiments.

### 2.3 Quantitative real-time polymerase chain reaction (qPCR)

Cells were lysed with Trizol reagent (Beyotime, China), followed by the addition of chloroform and gentle mixing to remove the organic phase. RNA was precipitated by adding isopropanol to the aqueous phase. The RNA pellet was washed with 75% ethanol and resuspended in TE buffer. RNA concentration was determined, and 2 μg of RNA was used for reverse transcription, utilizing the GoScript Reverse Transcription System (Promega, USA). SYBR qPCR Super Mix Plus (Takara, Japan) was employed for qRT-PCR according to manufacturer’s introduction. *GAPDH* served as the internal control. Relative RNA level was calculated relative to *GAPDH* gene expression using the 2^–ΔΔCT^ formula: ΔCT (test) = CT (target, test) – CT (ref, test); ΔCT (calibrator) = CT (target, calibrator) – CT (ref, calibrator); Target gene fold change = 2^– (ΔCT (test) – ΔCT^ (calibrator)). Primers used in this study are listed in (Supplementary Table S1).

### 2.4 Western blotting assay

Total proteins were collected using RIPA lysis buffer (Beyotime). After centrifuge at 12,000 rpm for 10 min, the supernatant was collected and separated in SDS-PAGE gel. The gel concentration was 12% when the molecular weight of target proteins was less than 50kd, while gel concentration was 10% if molecular weight more than 50kd. The proteins were transferred to PVDF membranes (Millipore), followed by blocking with 5% non-fat milk in TBS at room temperature for 1 h. The protein bands were then probed with primary antibodies (Proteintech) overnight at 4°C. Next day, the membranes were washed with PBST and hatched with HRP-conjugated anti-mouse or anti-rabbit antibody at room temperature for 1 h. The dilution factors of antibodies were determined by manufacturer’s introduction, usually 1:1,000. The bands were visualized after reaction with ECL reagent (Millipore). ImageJ software was used to analyze the gray value of the band and calculate the relative gray value of the target band (relative gray value = gray value/gray value of the reference band of the same sample).

### 2.5 Cell counting kit 8 (CCK-8) assay

The cells were collected after digestion and re-suspended to 5 × 10^3^ cells/100 µL as the initial plating density in complete media. Then 100 μL cell suspension was added to each hole of the 96-well plates, and the plate was placed in the 37°C cell incubator. After attachment, the media containing CYT (25 μM; MCE) and/or DOX (4 µM) were replaced and incubated for 24 h or the specified time. After that, 10 µL CCK-8 reagent (SolarBio) was added to each well, and the absorbance values at the wavelength of 450 nm were determined by microplate reader.

### 2.6 EdU assay

EdU experiment was conducted to examine cell proliferation. In short, cells were digested and seeded into 6-well plate. After attachment, the cells were treated with indicated drugs for 24 h, and then incubated with EdU working solution (SolarBio, 10uM) for 2 h without any positive controls. The cells were then washed with PBS and fixed for 15 min. After that, cells were reacted with Click solution for 20 min, washed with PBS, stained with Hoechst 33,342 (Beyotime) for 10 min. The Images were taken under a fluorescence microscope.

### 2.7 Colony formation assay

Cells were digested and suspended as single cells in complete medium and seeded into 6-well plate with 1,000 cells per well of 9.5 cm^2^. After incubation in 37°C incubator for 2 weeks, the colonies were stained with 2% crystal violet dye (in methanol) for 20 min. The cells were then washed with distilled water and air-dried. Images were taken by digital camera.

### 2.8 Wound healing

Cells were cultured in plates to form a tight monolayer. Drawing a straight line across the cell monolayer by a sterile scratch tool to form a “wound”. Cells were washed with serum-free culture medium to remove cell debris and non-adherent cells in the scratch. Cells were continued in culture and allowed to migrate to fill the scratch area. The healing of the scratch area was observed and recorded by microscope at different time points after the start of the experiment (0 h, 24 h, 48 h), and the distance of cell migration at different time points were compared.

### 2.9 Transwell assay

Cells were plated in the upper chamber with Matrigel coating for invasion assays, and the number of cells that migrated to the lower chamber was quantified after 24 h using crystal violet staining.

### 2.10 Cell apoptosis detection

Cell apoptosis was detected by Annexin V/PI apoptosis detection kit (Beyotime). In short, adherent cells were transfected and treated with indicated drugs, digested with enzyme without EDTA (Gibco), supplemented with complete medium, centrifuged at 1,000 rpm for 5 min, and the supernatant was discarded. Cells were then suspended with PBS that added with FITC-Annexin V reagent and PI reagent and incubated for 30 min. The samples were then examined by flow cytometer (BD Biosciences, USA). Data were acquired from a minimum number of events (at least 10,000 cells).and analysis was conducted using FlowJo software, wherein the populations of normal, early apoptotic, and late apoptotic cells were gated in the untreated group and these gates were directly applied to the treated group.

### 2.11 Xenograft tumor model

Balc/c nude female mice (Beijing Vital River Laboratory Animal Technology Co., Ltd.) that weighted about 20 g and aged 4–5 weeks old were housed in pathogen free environment for 1 week to acclimate. MDA-MB 231 cells were digested and resuspended in PBS at 1 × 10^8^/mL. A total of 100 μL cell suspension was injected subcutaneously to the right mammary gland. After the tumor size reached 100 mm^3^, the mice were randomly divided into three groups (Con/DOX/CYT + DOX)and given intraperitoneal injections of normal saline, Dox (3 mg/kg body weight), or CYT (15 mg/kg body weight) + Dox. Treatment was administrated every 3 days and tumor size was measured and calculated every 3 days and calculated as Volume (mm^3^) = 0.5 × (width^2^ × length). The mice were then succumbed to death by anesthesia injection, and tumors were collected and divided into two parts, one of which was fixed in 4% PFA (Thermo) and the other part was frozen in −80°C for protein and RNA measurement. All animal experiments were authorized by the Ethical Committee of Shandong Cancer Hospital (SDTHEC2021003102).

### 2.12 Immunohistochemical (IHC) staining

Tumor tissues were fixed, dehydrated in graded ethanol (Aladdin), and made into 5-µm slices. The tissue sections were heated in at 65°C and dewaxed in xylene and ethanol. After incubation with 3% H_2_O_2_ and 0.1% TritonX-100 (SolarBio), antigen retrieval was performed using sodium citrate. The samples were blocked in goat serum (Beyotime) and probed with anti-KI-67 antibody (Abcam) overnight at 4°C. Next day, the samples were hatched with secondary antibody for 1 h, and dyed with DAB reagent (Beyotime). The nuclei were stained with hematoxylin (Thermo). Images were taken with a microscope (Leica, Germany).

### 2.13 RNA sequencing analysis

The transcriptome sequencing samples were total RNA extracted from cells. RNeasy Mini Kit (250) Qiagen#74106 kit was used for sample RNA extraction in accordance with the kit procedures. The quality of obtained RNA samples was tested using Agilent Bioanalyzer 2,100 (Agilent technologies, Santa Clara, CA, US). Total RNA was quantified using Qubit^®^3.0 Fluorometer and NanoDrop One spectrophotometer. For cDNA library construction, the mRNA in the obtained RNA samples was separated and fragmented, and double-stranded cDNA was synthesized, the end of the strand was repaired and tailed, and the junction was added for enrichment. Then Qubit^®^ 3.0 Fluorometer was used to detect the concentration and Agilent 2,100 to detect the size of the library. Illumina NovaSeq 6,000 platform adopted for sequencing to generate raw data, which were processed to remove low-quality sequences, joint contamination, ribosome sequences (rRNA) to obtain high-quality sequences (Clean Reads). All subsequent analyses were based on Clean Reads. Clean Reads were compared to reference genes using Hisat2 software, FPKM was used to characterize different gene expression levels using Stringtie software, and genes were statistically quantified using edge software. Differentially expressed genes (DEGs) were then calculated with log2(FC) > 1 as the criterion. In order to determine the biological functions and signaling pathways of DEGs, we annotated each gene based on Gene Ontology (GO) and KEGG databases.

### 2.14 Statistical analysis

SPSS 20.0 and GrapgPad Prism 9.0 were used for statistical analysis, and the statistical differences among groups were tested by one-way ANOVA (single variance analysis). Paired Student’s t-test was used for the comparison of parameters between two groups. Data in each group were the average of three independent experiments, and the data were expressed as the mean ± standard deviation (SD). *P < 0.05 was considered as statistical difference.

## 3 Results

### 3.1 Cyperotundone stimulates the chemosensitivity of breast cancer cells *in vitro*

**FIGURE 1:**
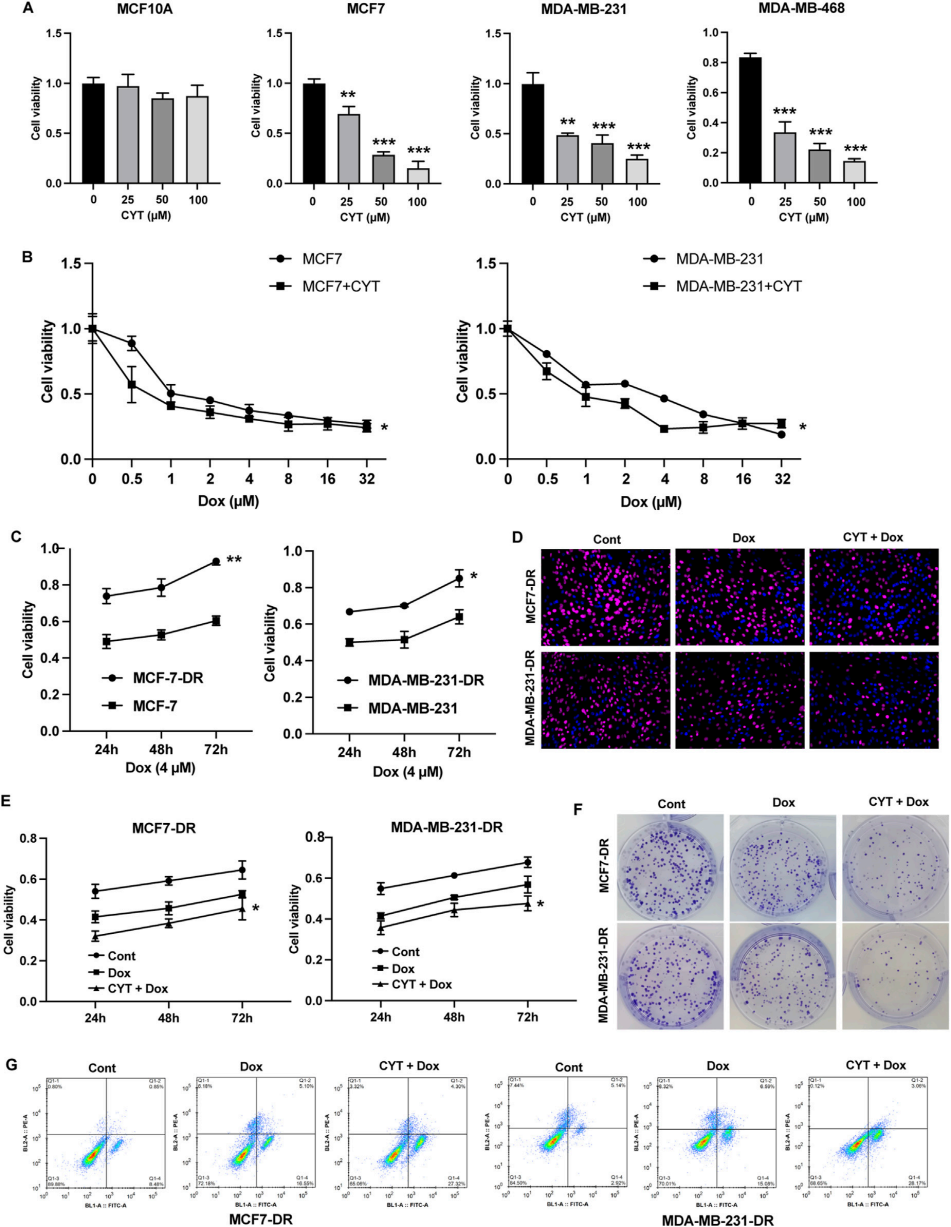
Cyperotundone stimulates the chemosensitivity of BC cells *in vitro*. **(A)** Effects of cyperotundone (CYT) on proliferation of normal mammary epithelial cells (MCF 10A) and breast cancer (BC) cells (MCF 7, MDA-MB-231 and MDA-MB-468). **(B)** Growth curve of MCF7 and MDA-MB-231 under doxorubicin (Dox, 0.1 µM) treatment. **(C)** Growth curve of drug resistant MCF7-DR and MDA-MB-231-DR compared with parental cells under Dox treatment. **(D–F)** Cell proliferation of MCF7-DR and MDA-MB-231-DR under treatment with CYT and Dox was measured by EdU, CCK-8, and colony formation assay. **(G)** Cell apoptosis was measured by flow cytometry. *P < 0.05, **P < 0.01, ***P < 0.001.

We first determined the effects of cyperotundone (CYT) on proliferation of cells and observed that CYT significantly inhibited the survival of breast cancer (BC) cells but not the normal breast epithelial cells MCF10A (Figure 1A). We observed that Doxrubicin (Dox) dose-dependently inhibited the survival of parental MCF7 and MDA-MB-231 (Figure 1B). Then, we evaluated the effects of CYT on drug resistance of BC cells using drug resistant MCF-7-DR and MDA-MB-231-DR cells (Figure 1C). The results of EdU (Figure 1D), CCK-8 (Figure 1E), and colony formation (Figure 1F) showed that compared with the Dox treatment group, combined treatment with CYT and Dox significantly inhibited cell growth and the proportion of EdU positive cells. Moreover, Dox treatment induced apoptosis of breast cancer resistant cells, which was significantly enhanced when combined with Epi (Figure 1G).

### 3.2 Cyperotundone suppresses metastasis and cancer stemness of drug resistant breast cancer cells

**FIGURE 2:**
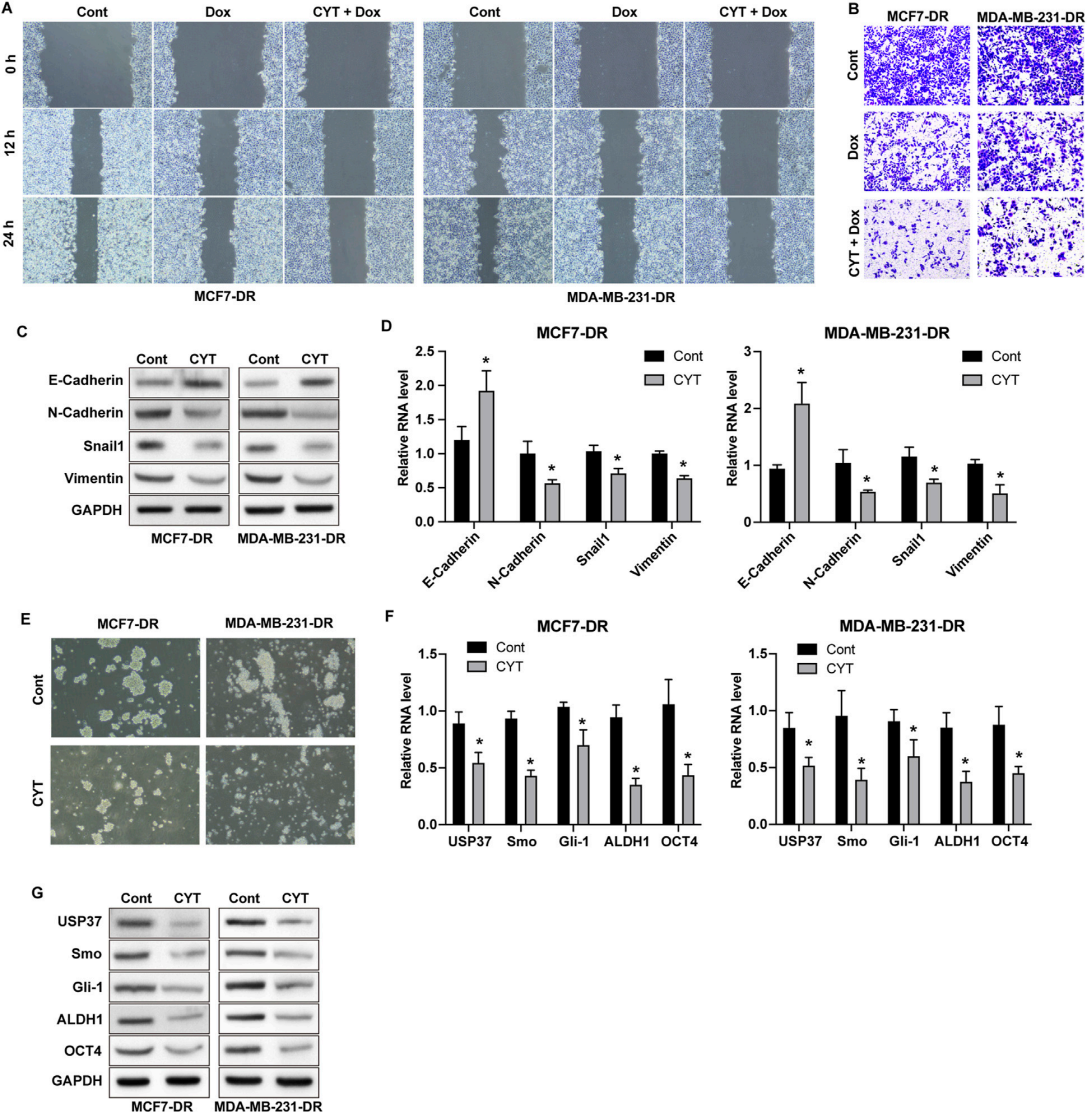
Cyperotundone suppresses metastasis and cancer stemness of doxorubicin resistant BC cells. MCF7-DR and MDA-MB-231-DR cells were treated with CYT and Dox. **(A)** Migration was detected by wound healing experiment. **(B)** Cell invasion and migration was determined by Transwell experiment. **(C)** Protein levels and **(D)** RNA levels of E-Cadherin, N-Cadherin, Vimentin, and Snail were determined by Western blot assay and qPCR assay. **(E)** Self-renewal ability of cells was detected by sphere formation assay. **(F)** Protein levels and **(G)** RNA levels of USP37, ALDH1, OCT4, Smo, and Gli-1 were determined by Western blot and qPCR assay. *P < 0.05, **P < 0.01.

The results from wound healing and Transwell assay showed that Dox treatment repressed the invasion and migration ability of drug-resistant BC cells, and combination of CYT and Dox further enhanced these effects (Figures 2A, B). The protein and RNA expression of N-Cadherin, Vimentin and Snail in MCF7-DR and MDA-MB-231-DR cells were notably downregulated, and E-Cadherin was upregulated by CYT treatment (Figures 2C, D). Moreover, the sphere formation ability of MCF7-DR and MDA-MB-231-DR cells were significantly repressed by CYT compared with control cells (Figure 2E), along with decreased RNA and protein levels of USP37, ALDH1, OCT4 and Smo/Gli-1 (Figures 2F, G).

### 3.3 Cyperotundone sensitize the *in vivo* anti-tumor effects of dox

**FIGURE 3:**
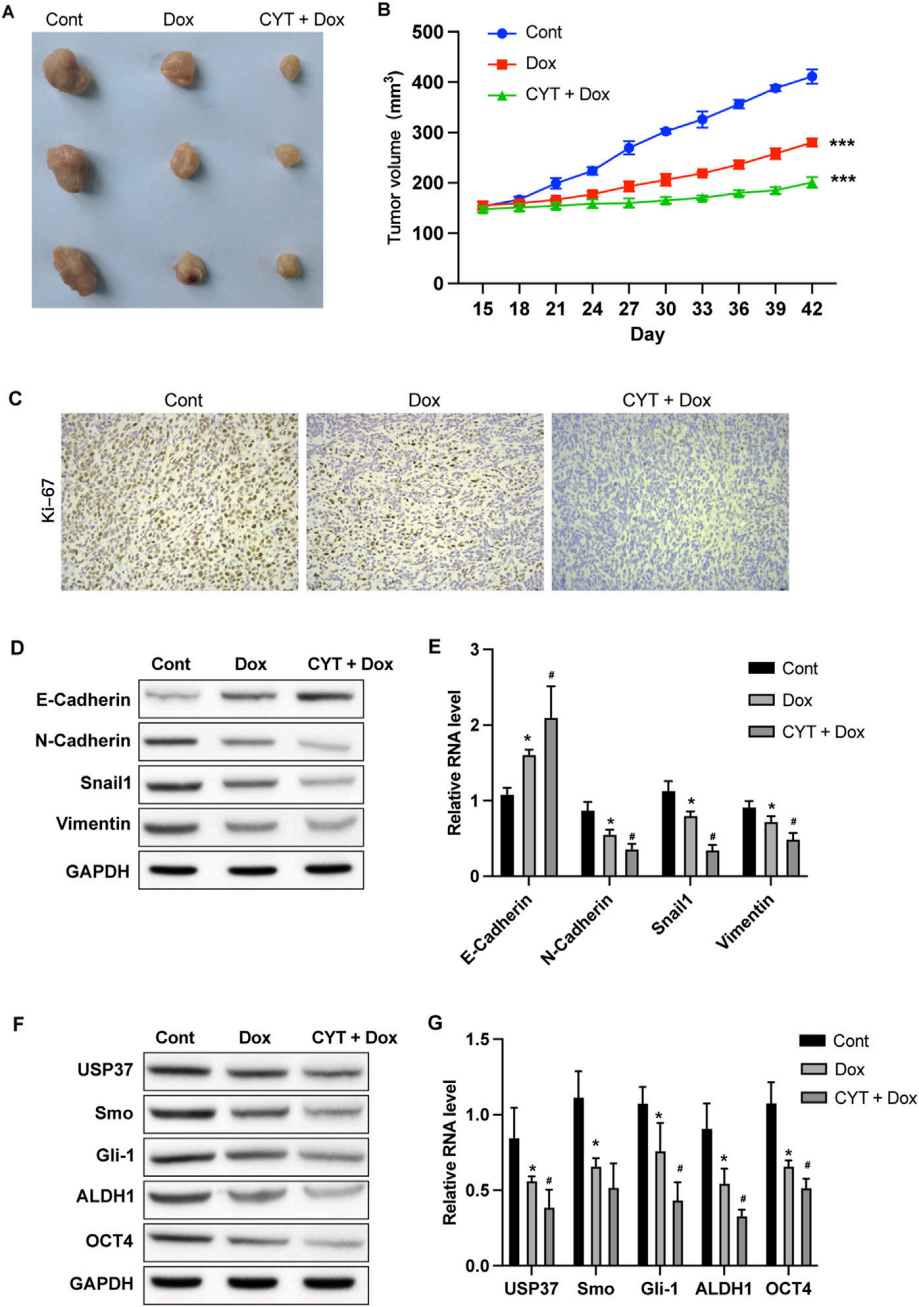
Cyperotundone suppresses the *in vivo* BC cell growth. Xenograft tumor model was established using MDA-MB-231 cells. **(A)** Image of xenograft tumors. **(B)** Tumor growth curve. **(C)** Expression of Ki-67 in tumor tissues. **(D)** Protein levels and **(E)** RNA levels of E-Cadherin, N-Cadherin, Vimentin, and Snail in mouse tumor tissues were determined by Western blotting and qPCR assay. **(F)** Protein levels and **(G)** RNA levels of USP37, ALDH1, OCT4, Smo, and Gli-1 in mouse tumor tissues were checked by Western blot and qPCR assay. *P < 0.05, **P < 0.01, ***P < 0.001.

We also established xenograft model using MDA-MB-231 cells to determine the *in vivo* effects of CYT on Dox sensitivity. Similar with the results from *in vitro* experiments, Dox treatment suppressed the *in vivo* growth of breast cancer cells and the tumor size, which was enhanced by combination with CYT (Figures 3A,B), simultaneously suppressed the KI-67 level (Figure 3C). Besides, by measure the RNA and protein level by qPCR and Western blotting, the expression of mesenchymal biomarkers (Figures 3D, E) and cancer stemness biomarkers (Figure 3F, G) was decreased in tumor tissues that treated with Dox, and CYT enhanced these effects.

### 3.4 Cyperotundone affects the growth, metastasis, and drug resistance of breast cancer cells via SRSF1

**FIGURE 4:**
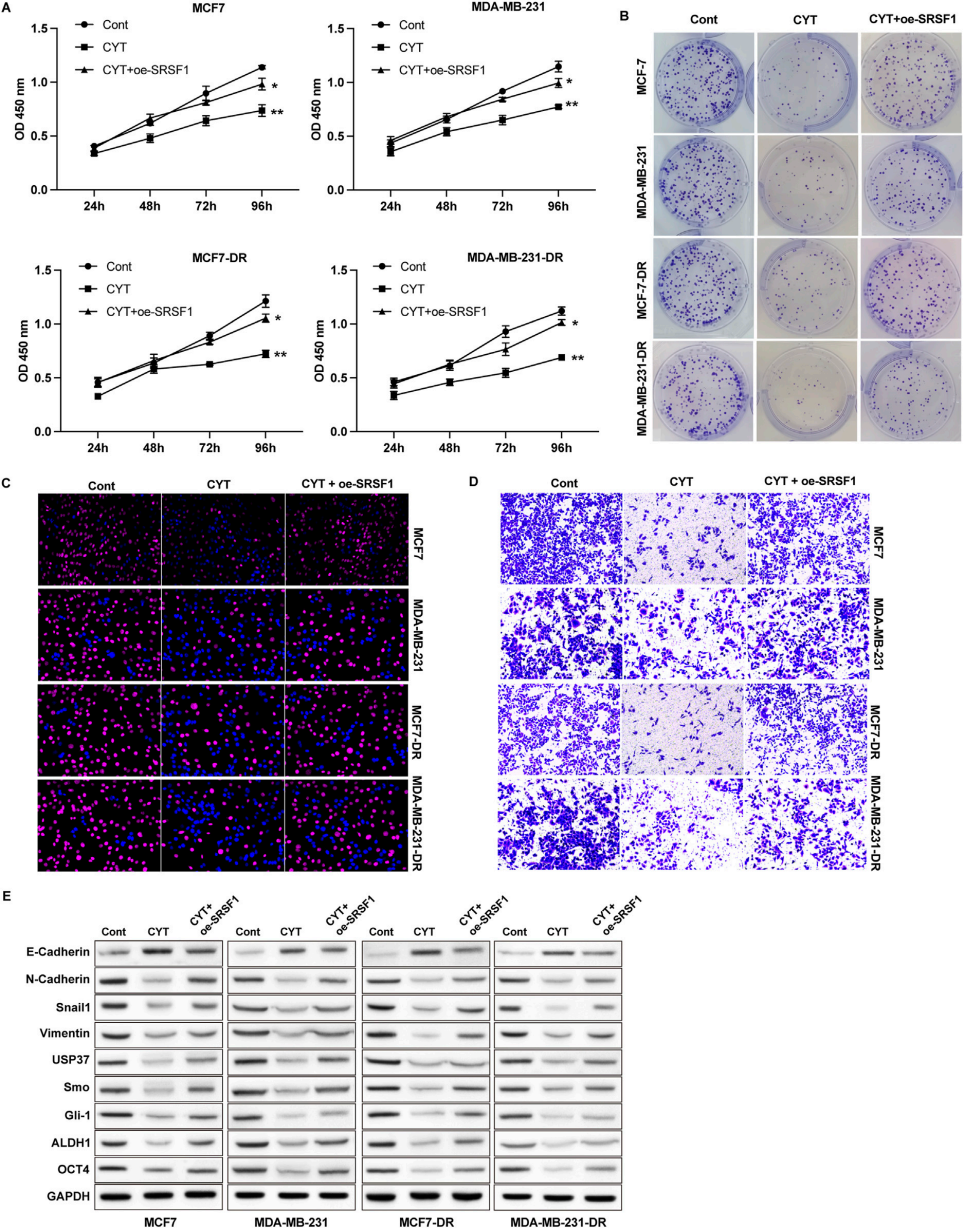
Cyperotundone affects the growth, metastasis, and doxorubicin resistance of BC cells (MCF 7-DR, MDA-MB-231-DR) via SRSF1. Cell proliferation was measured by **(A)** CCK-8, **(B)** colony formation and **(C)** EdU assay. **(D)** Cell invasion was measured by Transwell assay. **(E)** Protein levels of EMT and stemness biomarkers. *P < 0.05, **P < 0.01.

Next, we performed RNA sequencing analysis to determine the potential targets of CYT for drug resistance of BC cells. We performed GO and KEGG analysis on the differentially expression genes and identified that CYT treatment notably affected the genes that involved in regulation of uridine kinase activity, pre-mRNA binding, nucleotide phosphatase activity, protein synthesis, and cell cycle (Supplementary Figure S1A, B). We screened that the expression of SRSF1 and its potential downstream regulatory gene MYO1B are modulated by CYT treatment. Results from qPCR and Western blotting further verified decreased RNA and protein levels of SRSF1 and MYO1B in parental and doxorubicin resistant BC cells (Supplementary Figure S2A–C). Noteworthy, the expression of SRSF1 was notably higher in drug resistant BC cells compared with the parental cells (Supplementary Figure S2D, E). We next investigated the role of SRSF1 in CYT-treated by ectopic expression of SRSF1 in BC cells. We observed that overexpression of SRSF1 recovered BC cell growth curve (Figure 4A), colony formation ability (Figure 4B), and the EdU-positive cells (Figure 4C and Supplementary Figure S3A) that suppressed by CYT. Besides, the cell migration and invasion suppressed by CYT were notably enhanced by SFSR1 overexpression, as was shown in Transwell expression (Figure 4D). Consistently, the expression of mesenchymal biomarkers N-Cadherin, Vimentin and Snail and cancer stemness biomarkers USP37, ALDH1, OCT4 and Smo/Gli-1 in BC cells was suppressed by CYT and markedly recovered by SRSF1 overexpression (Figure 4E and Supplementary Figure S3B).

### 3.5 Cyperotundone modulates the growth of via SRSF1-regulated alternative splicing of MYO1B

**FIGURE 5:**
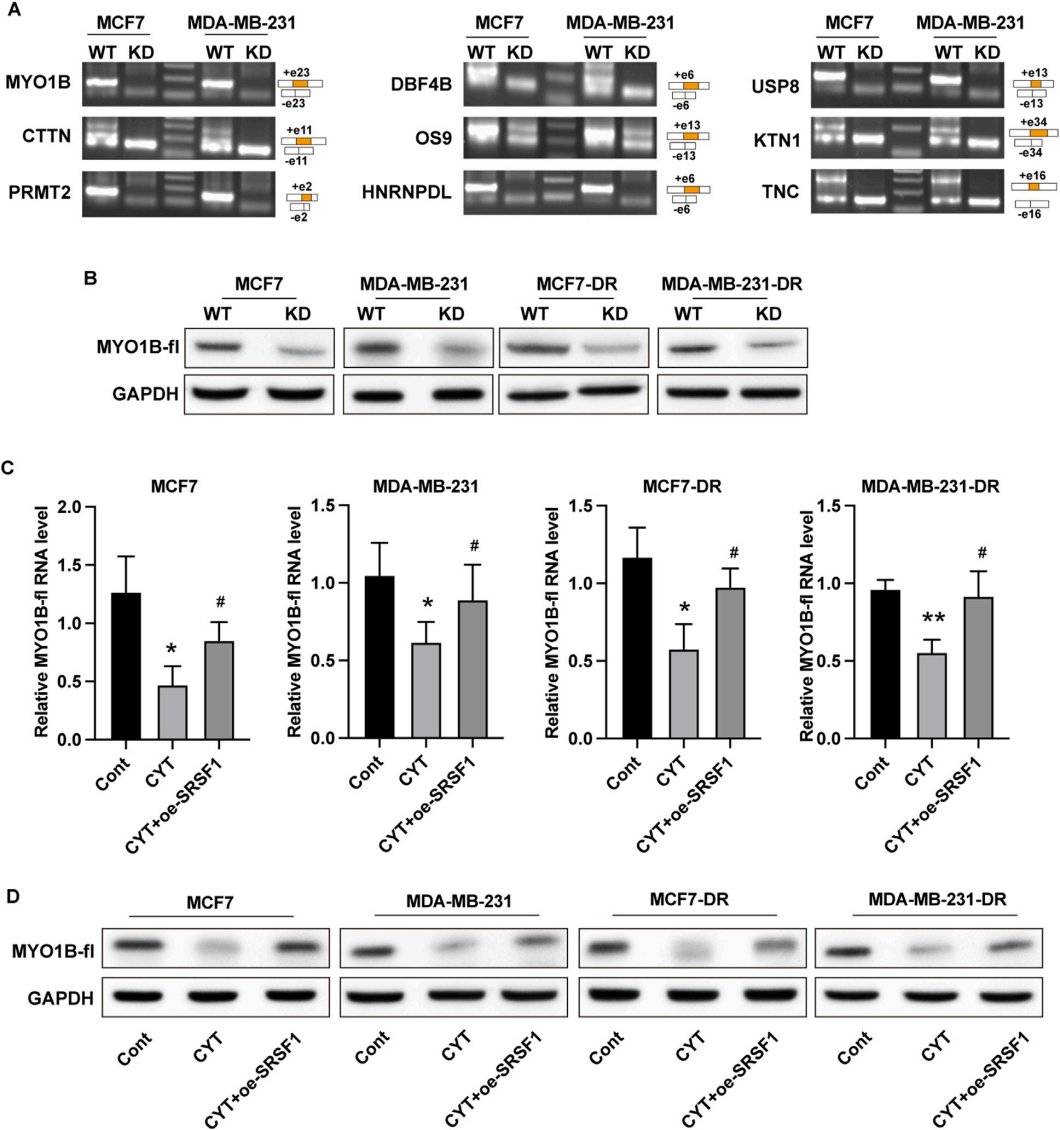
Cyperotundone regulates the alternative splicing effects of SRSF1 on MYO1B in parental (MCF 7, MDA-MB-231) and doxorubicin resistant (MCF 7-DR, MDA-MB-231-DR) BC cells. **(A)** The RNA levels of full length and alternative sliced transcript of targeted genes were detected by RT-PCR assay. **(B)** The expression of full length MYO1B was detected by Western blotting experiment. **(C)** The RNA levels of full length and alternative sliced transcript of targeted genes were detected by RT-PCR assay. **(D)** The expression of full length MYO1B was detected by Western blot experiment. *P < 0.05, **P < 0.01 vs. control; #*P* < 0.05, ##*P* < 0.01 vs. CYT group.

**FIGURE 6:**
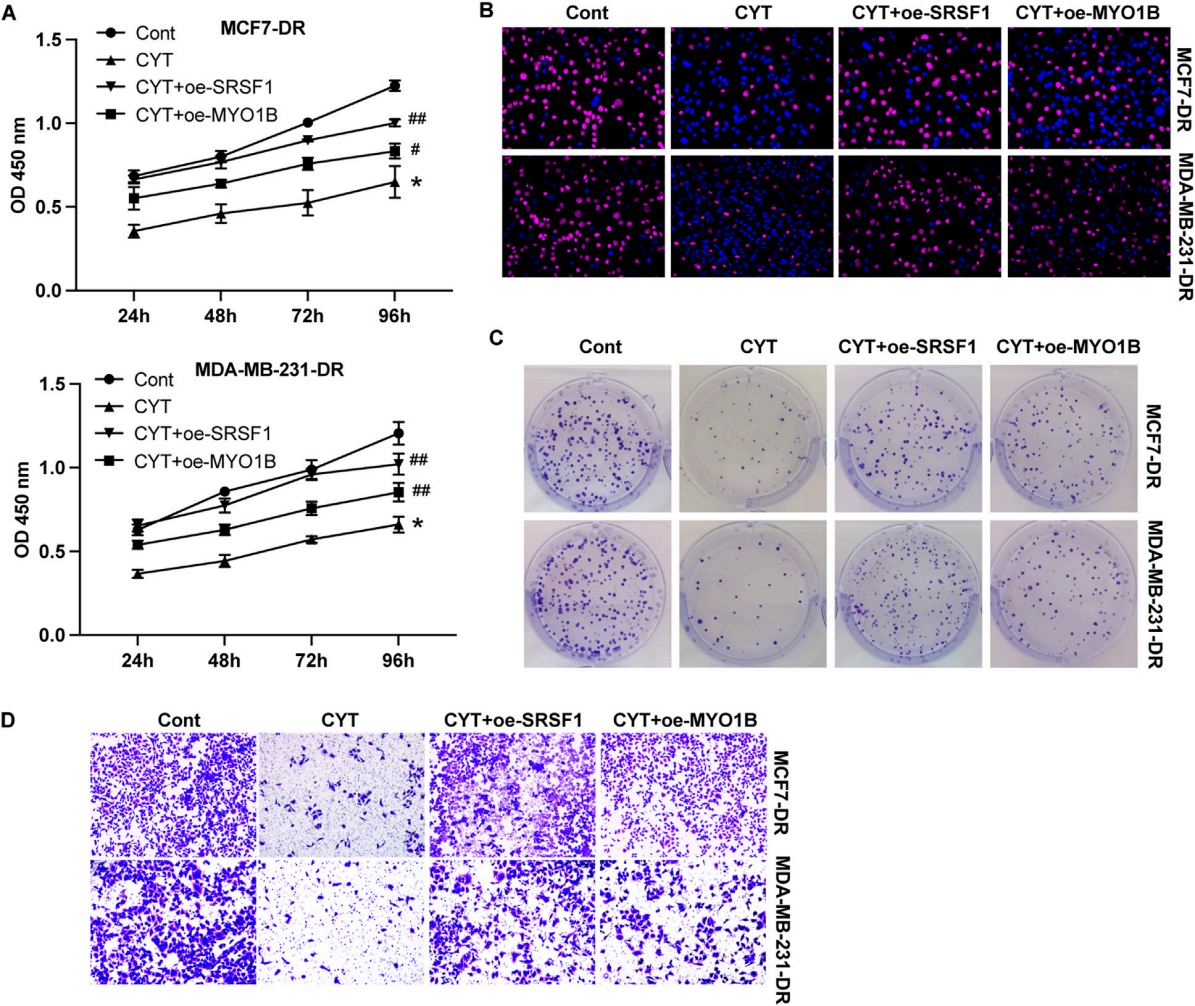
Cyperotundone affects doxorubicin resistant BC cell (MCF 7-DR, MDA-MB-231-DR) proliferation and metastasis via SRSF1/MYO1B axis. Cell proliferation was detected by **(A)** CCK-8 assay, **(B)** EdU assay, and **(C)** colony formation assay. **(D)** Cell invasion was measured by Transwell assay. *P < 0.05, **P < 0.01 vs. control; #*P* < 0.05, ##*P* < 0.01 vs. CYT group.

**FIGURE 7:**
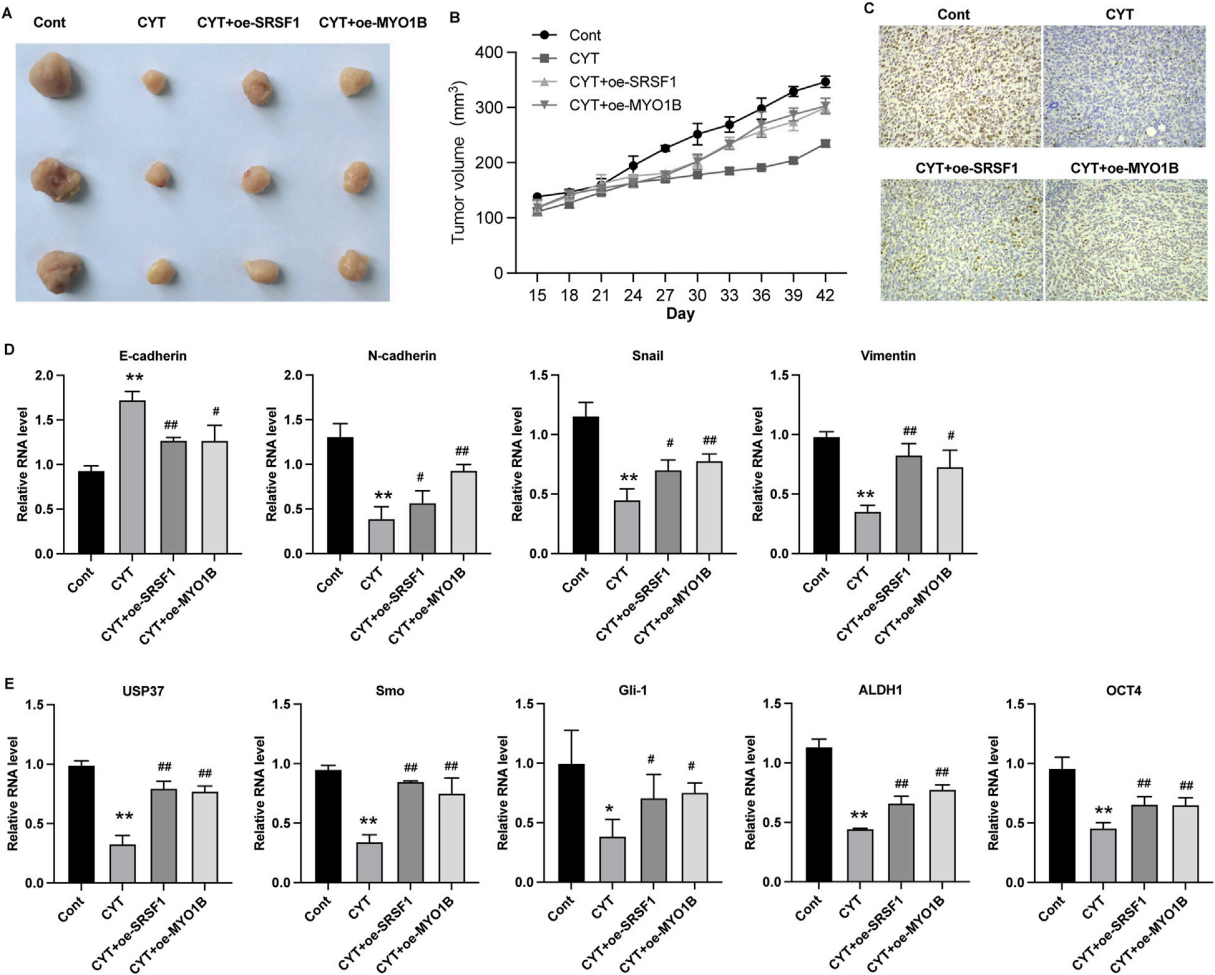
Cyperotundone inhibits the *in vivo* growth of BC cells (MDA-MB-231) through SRSF1/MYO1B axis. **(A)** Image of xenograft tumors in xenograft mouse model. **(B)** Tumor growth curve in xenograft mouse model. **(C)** Expression of Ki-67 in tumor tissues. **(D, E)** RNA level of E-Cadherin, N-Cadherin, Snail, Vimentin, USP37, Smo, Gli-1, ALDH1, and OCT4 in xenograft tumors. *P < 0.05, **P < 0.01 vs. control; #*P* < 0.05, ##*P* < 0.01 vs. CYT group.

SRSF1 plays a role in regulating alternative splicing in breast cancer cells. To determine whether SRSF1 is involved in the inhibitory effect of CYT on breast cancer cells by affecting alternative splicing, we examined several representative target genes. As shown in Figures 5A, B, the full-length MYO1B transcript (MYO1B-fl) level was decreased in SRSF1 depleted cells compared with the control cells, whereas the level of transcript with exon 23 depletion was elevated. Similarly, knockdown of SRSF1 can reduce the full-length transcript levels of CTNN, PRMT2, DBF4B, OS9, HNRNPDL, USP8, KTN1 and TNC (Figure 5A). Moreover, the expression levels of full-length MYO1B transcript (MYO1B-fl) and full-length protein in parental and drug-resistant BC cell lines were significantly decreased after CYT treatment, while SRSF1 overexpression could restore both levels (Figures 5C, D). These data suggested that CYT1 affects the SRSF1-regulated alternative splicing of MYO1B. Furthermore, the overexpression of SRSF1 or MYO1B could significantly restore the growth (Figures 6A–C) and invasion ability (Figure 6D) of MCF7-DR and MDA-MB-231-DR cells under the treatment of CYT *in vitro*. Similar with the *in vitro* results, the analysis by xenograft tumor model suggested that CYT notably reduced the tumor size (Figure 7A) and growth curve (Figure 7B) of MDA-MB-231-DR cells, along with decreased level of KI-67 (Figure 7C), whereas overexpression of SRSF1 and MYO1B reversed these effects. Compared with the control group, CYT notably downregulated the RNA levels of N-Cadherin, Snail, Vimentin, USP37, Smo, Gli-1, ALDH1, and OCT4, which were recovered by SRSF1 or MYO1B overexpression (Figures 7D, E).

## 4 Discussion

The emergence of chemotherapy resistance is one of the criticle reasons for cancer recurrence and poor prognosis of patients (Yao et al., 2019). Investigating therapeutic strategies that can alleviate chemotherapy drug resistance is crucial to improve the efficiency of breast cancer chemotherapy, which is a major clinical demand and an important scientific issue.

Some studies have reported the effects of extracts of Cyperus rotundus on tumors, such as 11,12-dihydroxy-4-ene-3-one, which demonstrated cytotoxic effects on ovarian cancer cells (Nogueira et al., 2020). Similarly, ethanol extracts of Cyperus rotundus have shown anticancer activity in triple-negative breast cancer (Wang et al., 2019). However, unlike these extracts, cyperotundone (CYT), a compound isolated from Cyperus rotundus, when combined with doxorubicin, not only induced apoptosis in tumor cells but also effectively inhibited chemotherapy-resistant breast cancer cells through inducing ROS production and NRF2/ARE signaling (Shao et al., 2023).

In our study, we found that CYT treatment significantly inhibited the proliferation of breast cancer cells and showed a dose-dependent trend but had no effect on the proliferation of normal breast epithelial cells. Moreover, administration of CYT notably suppressed the migration and cancer cell stemness, which was enhanced in CYT + Dox treatment. These experimental data suggesting that the preclinical activity of CYT is promising. However, the pharmacokinetic characteristics of CYT need to be further studied to explore the optimal human tolerated dose. Phase I clinical trials need to conduct to explore the side effects and appropriate dosage of CYT, and following phase II and III clinical trials to explore the efficacy and safety of CYT with Dox. The translation of our findings into clinical practice is possible through these standardized drug-development processes.

Alternative splicing is a widespread post-transcriptional regulation process of genes (Agosto and Lynch, 2018) (Bates et al., 2017). In recent years, it has been confirmed that it plays an important role in regulating the expression of oncogenes and tumor suppressor genes, and plays an important role in the occurrence and development of cancer (Agosto and Lynch, 2018). As an important alternative splicing modulator, SRSF1 could bind with different splicing regulatory elements to promote or inhibit splicing (Lv et al., 2021; Zhou et al., 2019). Several previous studies reported the important role of SRSF1-mediated alternative splicing in breast cancer (Xie et al., 2023; Kędzierska and Piekiełko-Witkowska, 2017; Yu and Fang, 2022; Anczuków et al., 2015). It has been reported that SRSF1 regulates alternative splicing events and harbors a binding motif involved in the splice switching of PTPMT1. By directly interacting with its motif in the exon 3 region, SRSF1 modulates this splice switching, partially contributing to its oncogenic function via the AKT/C-MYC axis. One study reported that long non-coding RNA HCG11 is downregulated in HR-positive breast cancer tissues and cell lines, and HCG11 could inhibit the malignant progression of breast cancer *in vivo* and *in vitro* (Xie et al., 2023). Mechanistically, HCG11 recruits SRSF1-targeted β-catenin and promotes its translation (Xie et al., 2023). Circular RNA RPAP2 was found to be downregulated in breast cancer samples and cell lines and correlated with the metastasis and TNM stage of breast cancer. Mechanistically, circRPAP2 could bind to SRSF1, which consequently inhibited SRSF1-mediated alternative splicing of PTK2, resulting in decreased levels of PTK2 mRNA and protein (Yu and Fang, 2022). While previous research has shown that SRSF1 overexpression is associated with increased malignancy and drug resistance in breast cancer, our results demonstrate that CYT treatment significantly reduces SRSF1 expression in drug-resistant cells (Song et al., 2024). SRSF1overexpression have been reported to involved in multiple resistance pathway. For example, CRNDE inducing cisplatin resistance through SRSF1/TIA1 signaling pathway in ovarian cancer (Wu et al., 2022). Circ_0001786 facilitates gefitinib resistance and malignant progression in non-small cell lung cancer via miR-34b-5p/SRSF1 (Ouyang et al., 2024). Notably, our data indicate that by targeting SRSF1-mediated alternative splicing of MYO1B, CYT can inhibit the chemo-resistance of breast cancer. The full-length MYO1B transcript (MYO1B-fl) level was decreased in SRSF1 depleted cells. The expression levels of full-length MYO1B transcript (MYO1B-fl) and full-length protein in drug-resistant BC cell lines were significantly decreased after CYT treatment, while SRSF1 overexpression could restore its levels. SRSF1 regulates the alternative splicing of MYO1B gene and inhibits its protein level, thereby inhibiting the proliferation, metastasis and stem cell properties of breast cancer cells, and promoting the chemosensitivity of drug-resistant breast cancer cells.

In clinical breast cancer samples, the expression of SRSF1 was upregulated and positively correlated with tumor grade, Ki-67 expression and poor prognosis. SRSF1 promoted the proliferation and migration of breast cancer cells and inhibited the apoptosis of breast cancer cells via regulating the alternative splicing of PTPMT1 (Du et al., 2021). In this study, through transcriptome sequencing analysis, we found that the SRSF1 level in breast cancer resistant cells was significantly reduced after receiving CYT treatment, and the cellular experiments verified this finding.

In addition, the expression levels of SRSF1 protein and RNA in drug-resistant breast cancer cells were significantly increased compared with parental cells, suggesting the SRSF1 may be the target of CYT in overcoming drug resistance. Knockdown of SRSF1 can reduce the expression level of full-length MYO1B protein in both drug-resistant and parental breast cancer cells, indicating that SRSF1 regulated the alternative splicing of MYO1B in breast cancer cells.

However, our study does have certain limitations. We only used two cell lines, MCF7 (HR positive BC cell) and MDA-MB-231 (TNBC cell), and found that CYT could overcome doxorubicin resistance. There are still many uncertainties from vitro findings into clinical practice, such as potential off-target effects of CYT, variability in preclinical models. Future research can be directed towards patient cohorts to help ascertain the correlation between the expression levels of SRSF1 and clinical outcomes, such as tumor grade, Ki-67 expression, and prognosis. Additionally, combination therapy approaches can be explored to investigate the concurrent administration of CYT with other anti-tumor agents, which may provide insights into its potential to overcome drug resistance and improve therapeutic efficacy. Our results only showed the effect of CYT in the above cell lines and indicated the potential effect in HR-positive breast cancer and triple-negative breast cancer. Further studies on HER2-positive breast cancer were also explored in our subsequent studies.

SRSF1 is a pivotal splicing factor that participates in the regulation of splicing processes, leading to a decrease in the expression of drug resistance-related proteins to enhance the sensitivity to chemotherapeutic agents. Thus, the development of small-molecule inhibitors or modulators targeting SRSF1 or MYO1B may provide a novel therapeutic strategy for drug-resistant breast cancer. Our findings identified the effects of CYT on identifying the potential regulatory with SRSF1/MYO1B axis via transcriptome analysis and experiment verification, supporting the anti-tumor effects of CYT against drug resistant breast cancer. Combining CYT with chemotherapeutic agents will enhance the uptake and sensitivity to chemotherapy, thereby achieving improved therapeutic efficacy.

## 5 Conclusion

In this study, we identified the effects of CYT on overcoming drug resistance of breast cancer and identified the potential regulatory with SRSF1/MYO1B axis via transcriptome analysis and experiment verification. Our findings supported the anti-tumor effects of CYT against drug resistant breast cancer and identified SRSF1 as a novel molecular mechanism.

## Data Availability

The data supporting this study are available in the GEO database (Accession Number: GSE291469), accessible via the following link: https://www.ncbi.nlm.nih.gov/geo/query/acc.cgi?acc=GSE291469.
