# Supplementary figures and images for: Cyperotundone promotes chemosensitivity of breast cancer via SRSF1
Source: Front Pharmacol. 2025 Mar 19;16:1510161. doi: 10.3389/fphar.2025.1510161 (PMC11961977; doi:10.3389/fphar.2025.1510161)

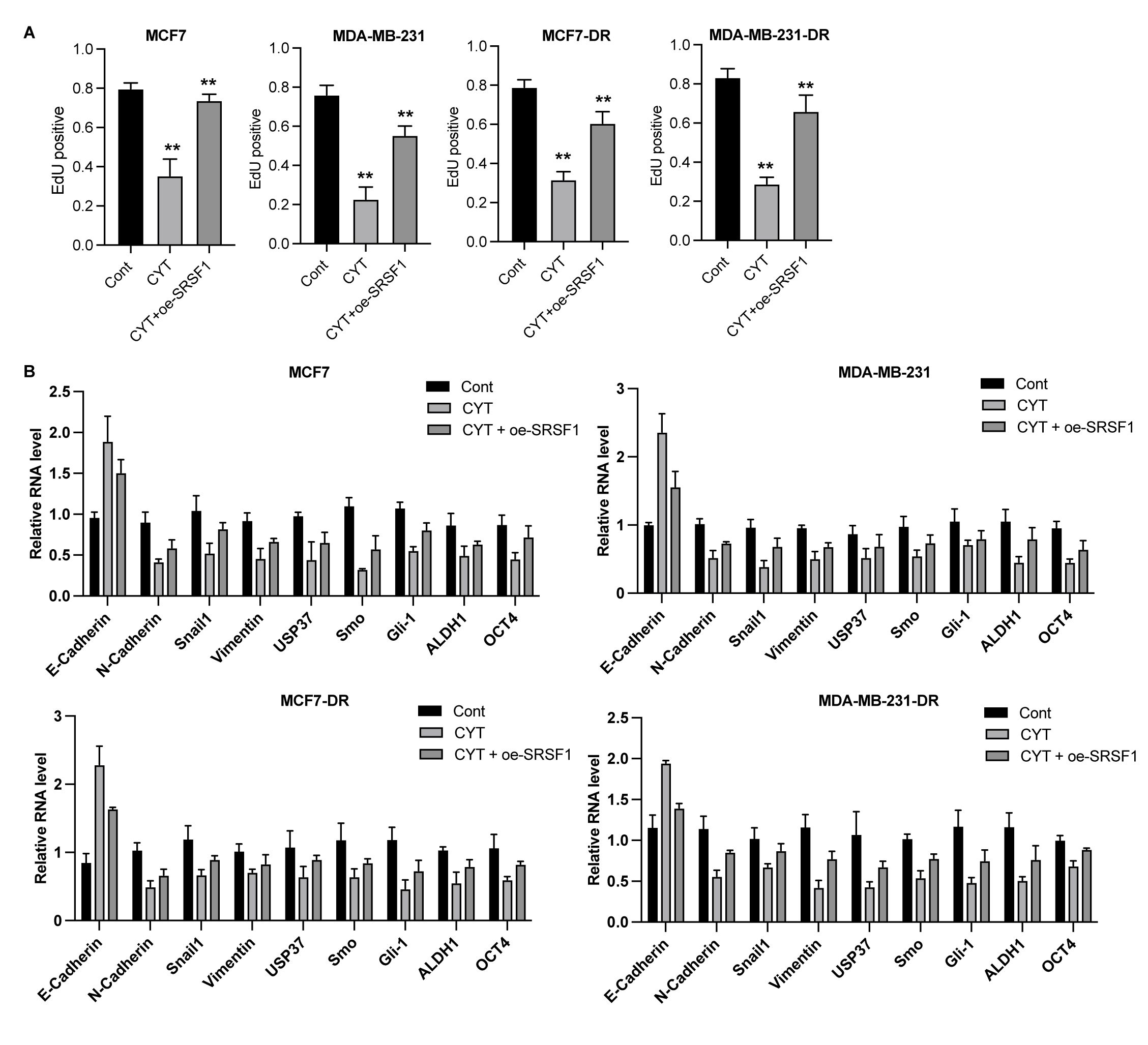

Supplement: Supplementary file 1 [file Supplementaryfile3.tif]

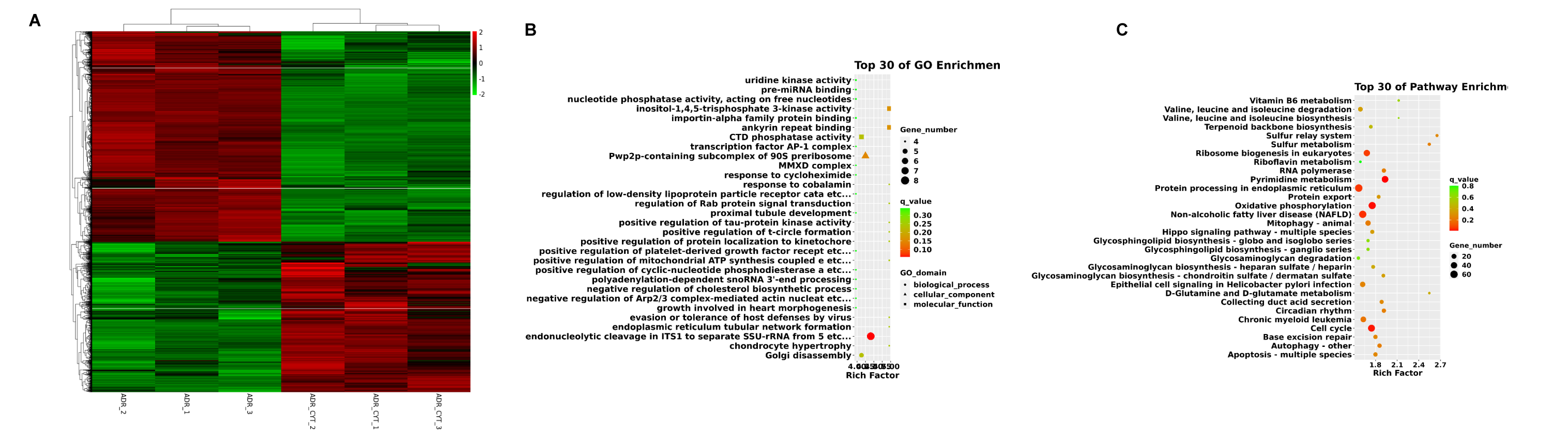

Supplement: Supplementary file 3 [file Supplementaryfile1.tif]

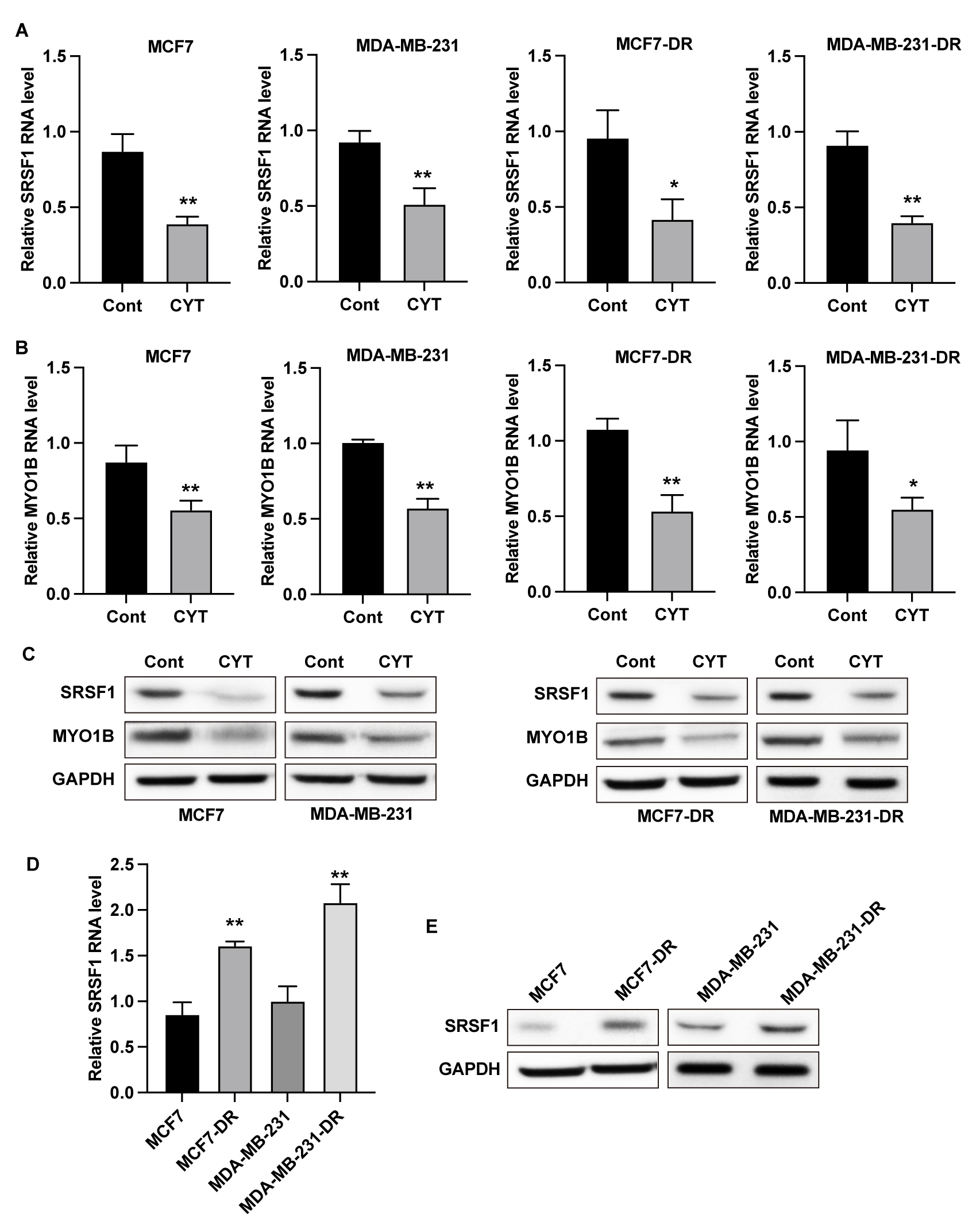

Supplement: Supplementary file 4 [file Supplementaryfile2.tif]
