# Supplementary material for: Cyperotundone promotes chemosensitivity of breast cancer via SRSF1
Source: Front Pharmacol. 2025 Mar 19;16:1510161. doi: 10.3389/fphar.2025.1510161 (PMC11961977; doi:10.3389/fphar.2025.1510161)
Supplement: Supplementary file 2 [file Table1.docx]

**Supplemental table S1 Primer sequence**

| Primer | Sequence |
| --- | --- |
| SRSF1-Forward | 5’- GCCGCATCTACGTGGGTAAC-3’ |
| SRSF1-Reverse | 5’- GAGGTCGATGTCGCGGATAG-3’ |
| E-Cadherin-Forward | 5’-CGAGAGCTACACGTTCACGG -3’ |
| E-Cadherin-Reverse | 5’- GGGTGTCGAGGGAAAAATAGG-3’ |
| N-Cadherin-Forward | 5’-AGCCAACCTTAACTGAGGAGT -3’ |
| N-Cadherin-Reverse | 5’- GGCAAGTTGATTGGAGGGATG-3’ |
| Snail-Forward | 5’- TCGGAAGCCTAACTACAGCGA-3’ |
| Snail-Reverse | 5’-AGATGAGCATTGGCAGCGAG -3’ |
| USP37-Forward | 5’- GGTCTGGTAGTTTTGGAGCCA-3’ |
| USP37-Reverse | 5’- GCAGAAGCCTGATTGTCTGAG-3’ |
| Gli-1-Forward | 5’- AACGCTATACAGATCCTAGCTCG-3’ |
| Gli-1-Reverse | 5’- GTGCCGTTTGGTCACATGG-3’ |
| ALDH1-Forward | 5’- GCACGCCAGACTTACCTGTC-3’ |
| ALDH1-Reverse | 5’-CCTCCTCAGTTGCAGGATTAAAG -3’ |
| OCT4-Forward | 5’- GGGAGATTGATAACTGGTGTGTT-3’ |
| OCT4-Reverse | 5’-GTGTATATCCCAGGGTGATCCTC -3’ |
| GAPDH-Forward | 5’- ACAACTTTGGTATCGTGGAAGG-3’ |
| GAPDH-Reverse | 5’- GCCATCACGCCACAGTTTC-3’ |
